# Label-free morphological sub-population cytometry for sensitive phenotypic screening of heterogenous neural disease model cells

**DOI:** 10.1038/s41598-022-12250-0

**Published:** 2022-06-16

**Authors:** Yuta Imai, Madoka Iida, Kei Kanie, Masahisa Katsuno, Ryuji Kato

**Affiliations:** 1 Department of Basic Medicinal Sciences, Graduate School of Pharmaceutical Sciences, Nagoya University, Tokai National Higher Education and Research System, Furocho, Chikusa-ku, Nagoya, Aichi 464-8601 Japan; 2 Department of Neurology, Nagoya University Graduate School of Medicine, Tokai National Higher Education and Research System, 65 Tsurumai-cho, Showa-ku, Nagoya, Aichi 466-8550 Japan; 3 Institute of Nano-Life-Systems, Institutes of Innovation for Future Society, Nagoya University, Tokai National Higher Education and Research System, Furocho, Chikusa-ku, Nagoya, Aichi 464-8601 Japan; 4 Department of Clinical Research Education, Nagoya University Graduate School of Medicine, Tokai National Higher Education and Research System, 65 Tsurumai-cho, Showa-ku, Nagoya, Aichi 466-8550 Japan; 5 Institute for Glyco-Core Research (iGCORE), Nagoya University, Tokai National Higher Education and Research System, Furocho, Chikusa-ku, Nagoya, Aichi 464-8601 Japan

**Keywords:** Data processing, High-throughput screening, Machine learning, Neurological disorders, Drug screening, Cellular imaging

## Abstract

Label-free image analysis has several advantages with respect to the development of drug screening platforms. However, the evaluation of drug-responsive cells based exclusively on morphological information is challenging, especially in cases of morphologically heterogeneous cells or a small subset of drug-responsive cells. We developed a novel label-free cell sub-population analysis method called “in silico FOCUS (in silico analysis of featured-objects concentrated by anomaly discrimination from unit space)” to enable robust phenotypic screening of morphologically heterogeneous spinal and bulbar muscular atrophy (SBMA) model cells. This method with the anomaly discrimination concept can sensitively evaluate drug-responsive cells as morphologically anomalous cells through in silico cytometric analysis. As this algorithm requires only morphological information of control cells for training, no labeling or drug administration experiments are needed. The responses of SBMA model cells to dihydrotestosterone revealed that in silico FOCUS can identify the characteristics of a small sub-population with drug-responsive phenotypes to facilitate robust drug response profiling. The phenotype classification model confirmed with high accuracy the SBMA-rescuing effect of pioglitazone using morphological information alone. In silico FOCUS enables the evaluation of delicate quality transitions in cells that are difficult to profile experimentally, including primary cells or cells with no known markers.

## Introduction

Advances in cell engineering technologies have contributed to the emergence of in vitro phenotypic screening as a leading drug screening method^1–3^. Label-free phenotypic analysis yields more information on live and intact cells than labeled phenotypic analysis^4–7^. The advantages of label-free image analysis are high-throughput screening, low cost, and non-invasive cell evaluation^7–16^. Additionally, label-free imaging enables the acquisition of transitional cellular information—during the culture period—instead of only end-point information. Compared with fluorescence imaging, label-free imaging is economical as it does not involve labeling, and this decreases the cost and time of analysis and simplifies the protocols. Furthermore, label-free imaging enables flexibility in performing assays as validation and optimization of staining (fluorescence wavelength overlap, bleaching effects, and transfection efficiency) are not required. The disadvantages of immunofluorescence image analysis involve the time-consuming optimization of specific and sensitive antibodies and probes and the quantification of selected bio-makers.

However, label-free image analysis also has some limitations. The implementation and data quality control of label-free image analysis can be challenging as the phenotypic information reflects a combination of biological outcomes. Consequently, the effectiveness of label-free image analysis is dependent on the type of data extracted from the image and the data extraction and processing method^7,17^.

Conventional label-free image analysis utilizes two types of image data utilization concepts. The first type involves the extraction of multiple features from the cells in the image to describe the morphological features of cells (area, roundness, and peripheral features^7,10–12,14–16^), intensity patterns, and textures^10,11,14,16–19^, which reflect the morphological characteristics observed in microscopy. Therefore, analysis results, especially the validity of the trained model, can be biologically interpreted. The second type involves the collection of small image tiles from the target area in the image as representative pixel patterns^9,13^. These tiles are used to develop training prediction models, such as deep learning models. Although the collected image tiles serve as functional descriptors, their features and usage in the model are not interpretable. We advocate the first concept, especially for “morphology-based features,” as the explanatory parameters used in machine learning models can be biologically interpreted^7,10–12,14–17^. Previously, we had reported that morphology-based features from single cells can be summarized to describe the cell “population profile.” These features are important for robust morphology-based quality predictions in mesenchymal stem cells^7,10,16,17^, induced pluripotent cells^11,14^, neuronal cells^15,20^, and myoblasts^12^. Thus, the total cell population is captured with multiple images under one culture condition. Although image analysis reveals population features through exhaustive cytometric measurements, targeting a specific sub-population using only morphological information has been challenging. Sub-population analysis is also challenging in cases of morphologically heterogeneous cells. Therefore, the use of label-free morphological analysis for evaluating heterogenic population data in drug screening is limited owing to the limitations associated with the analysis of cells exhibiting heterogeneous drug responses.

**Figure 1:**
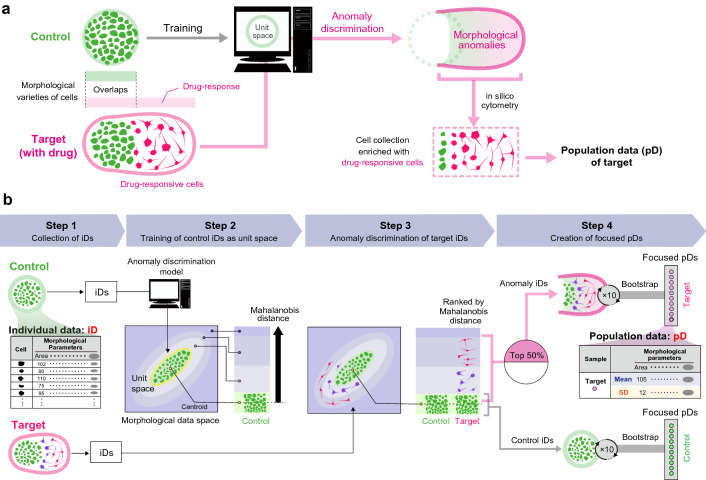
Schematic illustration of in silico featured-objects concentrated by anomaly discrimination from unit space (in silico FOCUS) analysis. (**a**) The concept of in silico FOCUS. This algorithm uses morphological variations in the control status for training the anomaly discrimination model and discriminates the “drug-responsive cells” as “morphological anomalies appeared in target status (with drug).” Collecting such morphological anomalies using in silico cytometry enables the collection of individual cell data with enriched drug-responsive cells to create population data (pD) to represent the target. (**b**) Steps in the data processing for in silico FOCUS. Step 1: individual data (iD) of cells, which are measured data of 14 morphological parameters, are collected. Step 2: Using only control iDs, unit space is trained for the anomaly discrimination model. In the anomaly discrimination model, unit space is placed in the center of morphological data space. The distance from the unit space is described by Mahalanobis distance. Step 3: Target iDs are ranked in the order of Mahalanobis distance by the discrimination model. Step 4: Using bootstrap, control-focused pDs are collected from total control iDs, while target-focused pDs are collected from anomaly iDs. All illustrations created by Adobe Illustrator 24.1.1 (https://www.adobe.com/jp/products/illustrator.html).

In this study, a novel label-free sub-population analysis method called “in silico FOCUS (in silico analysis of featured-objects concentrated by anomaly discrimination from unit space)” was developed. In silico morphological cytometric measurements focus only on the featured sub-populations (drug-responsive cells) in the total population (Fig. 1a).

In silico FOCUS, a data processing algorithm, can effectively collect information related to drug-responsive cells based on their morphological features using the anomaly discrimination concept. This algorithm automatically defines drug-responsive cells as “morphological anomalies that appear in drug response” by training the healthy morphologies in the control status. When the image from “target” (with drug) is used, this algorithm measures the Mahalanobis distance between individual data (iD) in “target” and “control” (unit space) and generates population data (pD) enriched with morphological anomaly data (anomaly iD; designated “focused pD”) to represent the target phenotype using morphological information alone (Fig. 1b). Thus, labeling or drug exposure experiments are not required to prepare training data for drug-responsive cell cytometry. As drug-responsive cells do not always represent most of the cell population and cannot be defined using stainable markers, the novel in silico FOCUS method enables cytometric analyses at the sub-population level based on cell morphology.

To investigate the performance of this method, the in vitro spinal and bulbar muscular atrophy (SBMA) cell models (AR-24Q and AR-97Q cells), which were previously developed by our group^21,22^, were used. However, the drug responses could not be effectively determined based on images alone owing to morphological heterogeneity.

SBMA, a neuromuscular disease, is characterized by muscle weakness, atrophy, and fasciculation of the limb and bulbar muscles^23,24^. Ligand-dependent toxicity of the aberrant androgen receptor (AR) protein—attributed to the presence of polyglutamine repeats—promotes motor neuronal degeneration^25,26^. Meanwhile, anti-androgen therapies involving leuprorelin acetate (a luteinizing hormone-releasing agonist) have been established based on the principle of inhibition of the AR-related hormone release cascade^27,28^. However, these therapies are associated with some adverse events, including sexual dysfunction and anti-anabolic effects on skeletal muscles^29^. Therefore, novel drug candidates with different mechanisms of action to mitigate AR-related dysfunctions must be identified. Target-based screening focused only on AR signaling can narrow down the search for potential drugs. Additionally, effective phenotypic screening technologies will enable the development of novel therapeutic strategies and targets and drugs for SBMA. In this study, the effectiveness and robustness of the in silico FOCUS method as a potential rapid phenotypic screening technique were examined to characterize and identify drug-responsive model cells.

## Results

### Morphological heterogeneity evaluation in model cells

To establish an effective and label-free phenotypic method for screening candidate drugs for SBMA, morphological heterogeneity in the SBMA model cells (healthy AR-24Q and diseased AR-97Q cells that harbor different poly-glutamine repeats in ARs) was quantified.

**Figure 2:**
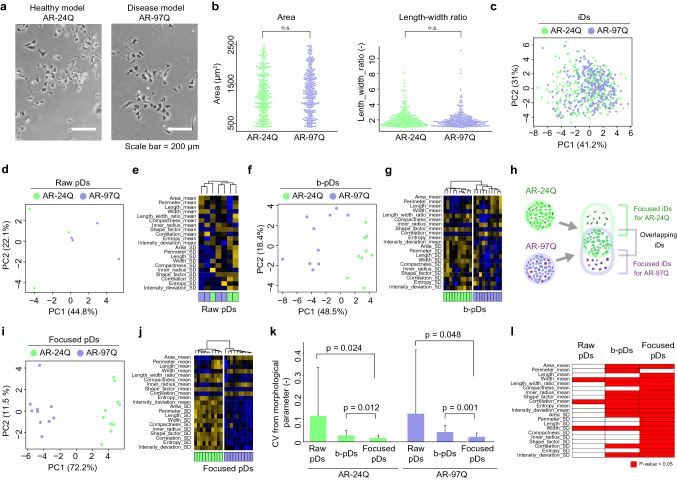
Evaluation of morphological heterogeneities and in silico featured-objects concentrated by anomaly discrimination from unit space (in silico FOCUS) analysis using model cells. (**a**) Representative image of model cells (healthy model, AR-24Q cells; disease model, AR-97Q cells). (**b**) Morphological distribution of 300 cells described by a single parameter. n.s. non-significant (N = 300). (**c**) Principal component analysis (PCA) of 100 cells described by 14 morphological parameters. (**d**, **e**) PCA and clustering of raw population data (raw pDs) (3 wells). (**f**, **g**) PCA and clustering of pDs with bootstrap (b-pDs) (N = 10). (**h**) Schematic illustration of in silico FOCUS applied to compare model cells. (**i**, **j**) PCA and clustering of pDs from in silico FOCUS (focused pDs;10 samples). (**k**) Evaluation of in silico FOCUS effect using variance of morphological parameters among pDs (Fig. S9a). *p* < 0.05 is indicated in the plot (N = 27). The bar plots indicate the mean of 27 coefficient of variations (CVs). Error bars indicate standard deviation. (**l**) Evaluation of in silico FOCUS effect using various morphological parameters that were significant (*p* < 0.05) between AR-24Q and AR-97Q (N = 10). All illustrations created by Adobe Illustrator 24.1.1 (https://www.adobe.com/jp/products/illustrator.html).

The results of the bulk assay (mitochondrial activity assay and metabolism measurement) on 20,000–30,000 cells revealed significant differences between model cell types even without AR stimulation (Fig. S1). However, these differences were undetectable in microscopy images examining < 100 cells per image (Fig. 2a). However, diverse morphologies were observed upon assessing 300 cells collected from replicated wells (Fig. 2b). Morphological analysis revealed that distinguishing AR-24Q and AR-97Q cells was difficult as the population was heterogenic and exhibited several overlapping morphologies. iD examination of single cells evaluated using principal component analysis (PCA) revealed that cells in each model type exhibit complex morphological heterogeneity in the multi-dimensional data space described using 14 morphological parameters. Thus, it was difficult to describe cell type characteristics (Fig. 2c).

Next, morphological analysis was performed using the population profile. The iDs were summarized as “population data (pD to represent each cell type (Fig. 2d–e). Consequently, the morphological characteristics of different cell types were distinctively identified as evidenced by distantly clustered plots and heatmaps. However, contamination of different cell types in the clusters with a heterogeneous heatmap pattern indicated that the summarized pDs were heterogeneous and consequently interfered with stable clustering.

### In silico FOCUS establishment for characterizing cells with high heterogeneity

To improve the stability of analysis, we study aimed to minimize the experimental bias that affects morphological information. Bias from “different fields-of-view” or “different wells” (Fig. S2) is prominently observed in case of morphologically heterogeneous cells. To mitigate this effect, the iDs were pooled in silico (Fig. S3). Although data pooling effectively minimized this bias, it reduced the data size that reflects morphological variety. Therefore, bootstrap was introduced to generate “pDs with bootstrap (b-pDs)” for statistical variation, which further improved the ability to distinguish the morphological differences between cell types (Fig. 2f–g). However, the heatmap pattern within the same cell type cluster indicated heterogeneous b-pDs. This suggested a high degree of heterogeneity in the cell population, which interfered with the generation of stable population data and clustering.

Next, this study aimed to enhance the morphological characteristics of each cell type and reduce “morphologically overlapping cells” from the pDs. An anomaly discrimination concept was introduced in the Mahalanobis-Taguchi (MT) method (Figs. 1b and S3)^30,31^. Drug-responsive cells with unique morphological characteristics (compared to the control cells) were defined as “anomaly iDs” based on the Mahalanobis distance. To enhance the effect of anomaly iDs in the pDs, the top 50% of iDs ranked in the order of Mahalanobis distance were acquired. In this concept, threshold need not be considered for anomaly iD definition. Thus, an increased number of morphological changes occurring due to drug response leads to an increased number of pDs from in silico FOCUS (= focused pDs) comprising anomaly iDs.

To demonstrate the effectiveness of FOCUS in silico, cell type discrimination was evaluated using focused pDs. Conceptually, cell-specific anomaly iDs were concentrated on, and the number of cells with major overlapping morphological features was minimized using in silico FOCUS (Fig. 2h). The differences in morphological characteristics between the cell types when analyzed based on focused pDs (Fig. 2i–j) were found to be more distinct than the differences observed on analysis based on raw pDs (Fig. 2d–e) and b-pD (Fig. 2f–g). The pattern of focused pDs in the heatmap was homogeneous, indicating that in silico processing of FOCUS data effectively enhanced the stability of phenotypic evaluation. Comparative analysis of the coefficient of variations (CVs) among pDs in all morphological parameters revealed that the large variance reflecting the experimental bias in raw pDs decreased by more than fivefold in focused pDs, which were significantly more stable than b-pDs (Fig. 2k). In focused pDs, the number of significant morphological parameters (AR-24Q vs. AR-97Q cells) increased (Fig. 2l). Hence, in silico processing of FOCUS data effectively generated stable pDs and clusters.

### Dihydrotestosterone (DHT)-responding phenotype analysis in model cells

Next, the ability of in silico FOCUS to identify drug-responding phenotypes in model cells was examined. DHT, which was selected to induce characteristic phenotypic changes observed in SBMA^21,22,26^, is an AR ligand that induces disease phenotype in AR-97Q cells by promoting pathogenic nuclear AR accumulation and cell death through transcriptional dysregulation. The physiological AR cascade activates AR-24Q cells without any detrimental effects.

**Figure 3:**
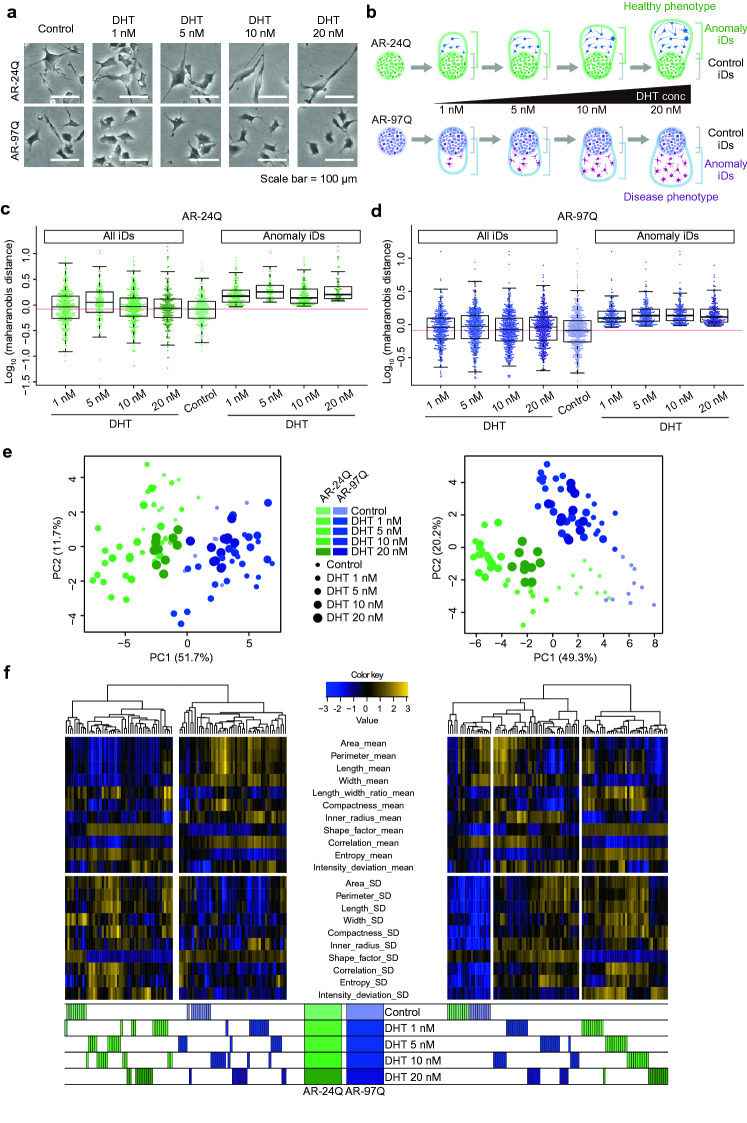
Evaluation of phenotypic responses of model cells to dihydrotestosterone (DHT). (**a**) Representative images depicting the effect of DHT on model cells. (**b**) Schematic illustration of in silico featured-objects concentrated by anomaly discrimination from unit space (in silico FOCUS) analysis used to compare the phenotypic response of model cells to DHT. (**c**, **d**) Distribution of Mahalanobis distances of individual data (iDs) (all iDs, control, and anomaly iDs from in silico FOCUS) from the centroid of the unit space defined by control iDs. Box plot (median at the center, and the 25 and 75 percentiles at the end) with whiskers (standard deviation) overlaid on the beeswarm plot. The red line indicates the mean of control iDs. (**e**, **f**) Principal component analysis (PCA) and clustering of population data (pD) with bootstrap (b-pDs) (left) and pDs from in silico FOCUS (focused pDs) (right). All illustrations created by Adobe Illustrator 24.1.1 (https://www.adobe.com/jp/products/illustrator.html).

Visual analysis of DHT stimulation response revealed that healthy cells elongated to a greater degree than diseased cells (Fig. 3a). However, distinguishing between control and DHT-responding conditions was found to be difficult upon comparing the length_width_ratio distribution of 300 cells from each condition (Fig. S4a).

To emphasize the morphological characteristics of the DHT-responding cell sub-population, in silico FOCUS was used to concentrate anomaly iD in focused pD from each model cell (Fig. 3b). An increased number of anomaly iDs was expected to increase the extent to which the focused pDs would reflect the drug response, even from a small sub-population. Consistently, the distribution of anomaly iDs exhibited a higher degree of deviation than the control population distribution and a lower degree of deviation than that of iDs without in silico FOCUS (Fig. 3c,d).

Comparative analysis of the morphological characteristics using PCA revealed that the morphological response to DHT in focused pDs was more distinct than that in b-pDs in both model cell types (Fig. 3e). The b-pDs clustered based on the cell type, whereas the focused pDs indicated a phenotypic transition of clusters starting from the control phenotype to the DHT-responding phenotype. The positions of these drug-responding clusters in PCA indicated that the AR-24Q and AR-97Q cells exhibited characteristic morphologies of drug-responding cells.

The effect of in silico FOCUS was visualized using clustering (Fig. 3f). Heterogeneous heatmap patterns within clusters related to different conditions indicated that the b-pDs were not stable to characterize the DHT responses as differences based on cell type were too prominent to allow this characterization. In contrast, the focused pDs allowed for homogenous clusters. The clustering results also indicated characteristic transitions in the morphologies of responder cells based on DHT concentrations in both AR-24Q and AR-97Q cells. The distinctly clustered AR-24Q data perfectly reflected the DHT dose–effect and indicated that the DHT responses of healthy cells could be extracted using in silico FOCUS analysis. The control status of the non-responders also indicated that the focused pDs were sensitive to morphological differences even at a DHT concentration of 1 nM.

Statistical analysis of heterogeneity in morphological parameters revealed that the variance of morphological parameters (= CV from morphological parameters) in focused pDs was significantly lower than that in b-pDs with DHT responses (Fig. S4b). Moreover, the average proportion of significant morphological parameters between control and. target groups increased from 46% (b-pDs among both cell types) to average 76% (focused pDs among both cell types) (Fig. S4c).

Furthermore, the phenotypic characteristics of cells could be interpreted from the clustering of the focused pDs. The “mean of length-related parameters” (such as length_width_ratio, perimeter, and compactness) increased after DHT administration (till 10 nM concentration), while the “mean of inner_radius” remained less in AR-24Q cells (Fig. 3f). This response indicates that compared with those in control cells, the elongation was higher and the morphology was thinner in DHT-stimulated healthy cells. However, the increase in “standard deviation (SD) of size-related parameters” (such as area, perimeter, and inner radius) suggested that this elongation was highly heterogeneous. Although the morphological characteristics of AR-97Q cells were not as distinct as those of AR-24Q cells, similar increases in “SD of size-related parameters” were detected. This indicated increased morphological heterogeneity in both cell types. The increased “mean of inner_radius and intensity_deviation” in the AR-97Q cells indicated that DHT promoted heterogenous cell morphology with decreased elongation. These findings suggest that the neural model cells were morphologically heterogeneous, a phenomenon that increased with drug response.

As in silico FOCUS collects top “anomaly iDs” from the target cell population relative to the control cell population, these anomaly iDs can be generated only by chance. To verify that the anomaly detection effect of in silico FOCUS is not affected by false-positive anomalies, “control cell population” was examined for both control and target groups. Conceptually, the extracted populations A and B should never be completely the same even if they are obtained from the same cell population. Therefore, anomaly iDs can be forcibly generated. However, the efficacy of in silico FOCUS with respect to detecting the drug responses will not be overcome if such pseudo-anomalies cannot form meaningful focused pDs. These results indicated that the pseudo-focused pDs can be forcibly generated but they do not disturb the cluster of real focused pDs reflecting the DHT effect (Fig. S4d). Additionally, this validation of in silico FOCUS data using established positive control is important.

In silico FOCUS is a type of image cytometry analysis that generates focused pD by summarizing iDs. Hence, the sample size of iD collection can markedly affect the robustness of analysis. The effect of iD collection size (Table S1) starting from the default collection number (100 iDs) was investigated. The efficacy of in silico FOCUS with respect to evaluating the drug effect markedly decreased when iD collection was reduced. Hence, more than 100 iD collections are essential.

In summary, in silico FOCUS analysis was found to exhibit robust performance with respect to the evaluation and interpretation of heterogenic cellular responses upon using the control drug.

### Pioglitazone (PG)-responding phenotype evaluation in model cells

Next, the ability of in silico FOCUS analysis to effectively evaluate rescue responses in model cells was examined. PG exerts therapeutic effects on SBMA model cells (AR-97Q stimulated with 10 nM DHT) and murine models by activating the expression of PPARγ^21^. Based on the results obtained in previous studies, cells treated with PG at concentrations > 0.1 µM were used as positive controls and were defined as exhibiting a “rescued phenotype.”

**Figure 4:**
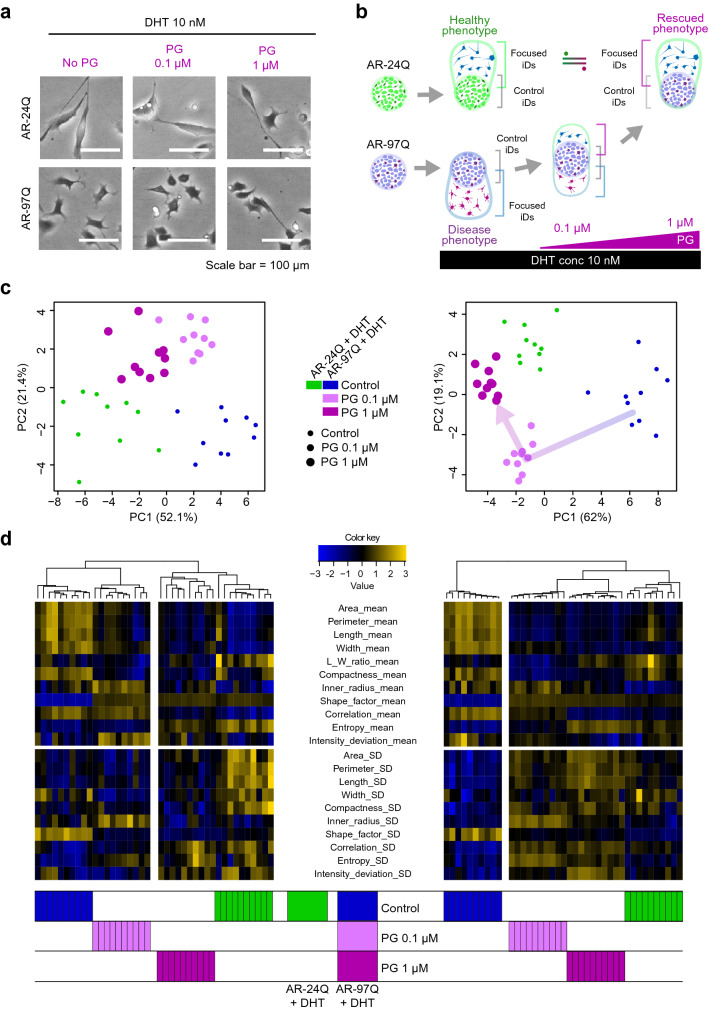
Evaluation of phenotypic responses of dihydrotestosterone (DHT)-stimulated model cells treated with pioglitazone (PG). (**a**) Representative images depicting the effect of PG on the (DHT)-stimulated model cells. (**b**) Schematic illustration of in silico featured-objects concentrated by anomaly discrimination from unit space (in silico FOCUS) analysis used to compare the phenotypic response of the model cells to PG. (**c**, **d**) Principal component analysis (PCA) and clustering of population data (pDs) with bootstrap (b-pDs) (left) and pDs from in silico FOCUS (focused pDs) (right). PCA of focused pDs; the arrow indicates the dose-dependent morphological rescue effect of PG. All illustrations created by Adobe Illustrator 24.1.1 (https://www.adobe.com/jp/products/illustrator.html).

The PG rescue effect was reflected in AR-97Q cell morphology (Fig. 4a). AR-97Q cells, which exhibit a shrunken morphology, appeared elongated after PG treatment (1 µM). However, significant differences between control and PG-treated cells were difficult to detect when 300 cells were analyzed (Fig. S5a). Thus, morphological heterogeneity in the model cell made it difficult to detect the rescue effect using a simple method. Therefore, in silico FOCUS was expected to classify the rescued phenotype in AR-97Q cells based on their similarity to healthy AR-24Q cells (Fig. 4b).

Next, in silico FOCUS analysis was used to determine its ability to detect the subtle drug responses of a small cell population. The anomaly iD distribution indicated that in silico FOCUS could differentiate these cell sub-populations from control cells (Fig. S5b). From the representative morphologies of iDs, their elongated morphology can be confirmed, especially in anomaly iDs with large Mahalanobis distances (Fig. S6).

PCA visualization with both b-pDs and focused pDs indicated that PG treatment mitigated the pathological morphological changes in AR-97Q cells (Fig. 4c). However, only analysis of focused pDs enabled the detection of a dose-dependent relationship for this morphological transition. SpecificallyFocused pDs clustered close to the healthy phenotype at PG concentrations > 0.1 µM. The significant decrease in CV from morphological parameters indicated that focused pDs exhibited significantly higher stability than b-pDs (Fig. S5c).

The clustering results also confirmed the effectiveness of the in silico FOCUS method (Fig. 4d). The homogeneity of heatmap patterns under the same condition cluster in focused pDs was markedly higher than that in b-pDs, indicating the stable clustering performance of pDs (Fig. 4d). Although the main cluster branches that distinguish “disease phenotype” or “healthy phenotype” were similar, only focused pDs formed both PG-responding morphology clusters (with both 0.1 and 1 µM) close to the healthy phenotype cluster. The heatmap pattern in the cluster indicated gradual morphological recovery, reflecting PG dose–response. Clustering stability was also evidenced by the increase in morphological parameters in the focused pDs relative to b-pDs, showing significant control vs. target differences (Fig. S5d). Based on the interpretation of parameters (Fig. 4d), the increase in “mean of length-related parameters” supported the hypothesis that PG treatment recovers the thin and elongated morphology in AR-97Q cells.

To confirm that the efficacy of in silico FOCUS for detecting the PG rescue effect is not affected by false-positive anomaly iDs generated only by chance, the focused pDs from PG-responding conditions were compared with the pseudo-focused pDs forcibly generated using control status for “target” (Fig. S5e). Comparative analysis revealed that pseudo-focused pDs do not disturb the profile of real focused pDs upon PG treatment.

These findings indicate that the in silico FOCUS method can effectively detect drug rescue in morphologically heterogeneous SBMA cells. Additionally, this method may help determine the effective drug concentration based on morphology data alone.

### Phenotype classification model development for drug screening

Unsupervised analysis indicated that the focused pDs from in silico FOCUS were effective descriptors for identifying the characteristic phenotypes in the model cells. This prompted us to further develop supervised machine learning models for automatically classifying the phenotypes to enable high-throughput image-based drug screening. This involves screening drug candidates using AR-97Q cells and evaluating their drug-responsive morphology using the phenotype classification models.

**Figure 5:**
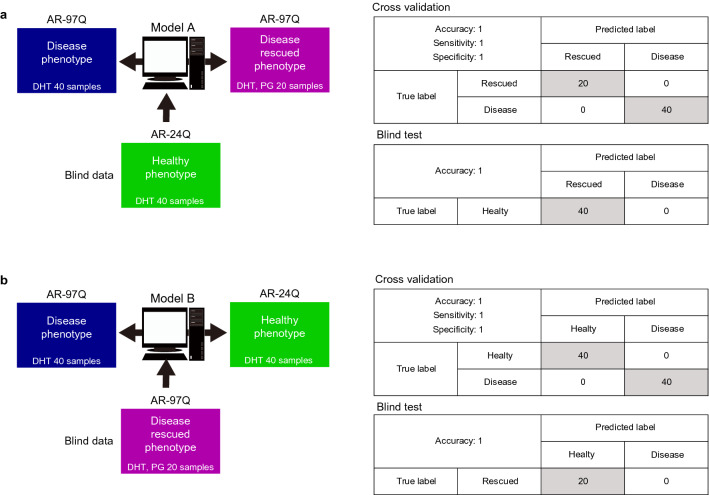
Concept and performances of phenotype classification trained with focused population data (pDs). (**a**) Model A is trained to classify the disease phenotype (AR-97Q cells with dihydrotestosterone (DHT)) and the rescued phenotype (AR-97Q cells treated with pioglitazone (PG) and DHT). The classification performances based on cross-validation and blind test data are shown in tables. (**b**) Model B is trained to classify the disease phenotype and healthy phenotype (AR-24Q cells with DHT). The classification performances based on cross-validation and blind test data are shown in tables. All illustrations created by Adobe Illustrator 24.1.1 (https://www.adobe.com/jp/products/illustrator.html).

Model cells exhibited the following two phenotypes that could be trained as “hit” in the phenotype classification: the “rescued phenotype of AR-97Q” (indicating the PG effect) and the “healthy phenotype of AR-24Q” (served as the control). The following two classification models were constructed: model A, trained with “disease” and “rescued”; model B, trained with “disease” and “healthy” (Fig. 5). Both models exhibited high classification accuracy in cross-validation. However, model A could be over-fit to recognize only the PG effect in AR-97Q cells, while model B could be over-fit to recognize only cell type differences. Therefore, model A with “healthy” and model B with “rescued” as blind test data were tested to confirm their performance universality. Both models perfectly predicted the blind test data as “hit,” indicating that these are over-fit narrow models for phenotype classification. Thus, the morphological profile (focused pD) extracted using in silico FOCUS was effective for constructing high-accuracy and robust image-based classification models.

## Discussion

In this study, we developed an effective in silico image cytometry method (in silico FOCUS) using the anomaly discrimination concept to sensitively evaluate phenotypes in SBMA model cells exhibiting morphological heterogeneity. Processing of in silico FOCUS data enabled the extraction of delicate morphological drug responses of a minor sub-population of model cells as stable “focused pD” to enable robust morphological analysis of drug effect based on morphological information alone. To the best of our knowledge, this is the first study to exclusively employ morphological sub-population analysis for SBMA drug screening and discuss the importance of in silico data processing based on the morphological profile at the population level with a reduced experimental bias for robust label-free image analysis. As in silico FOCUS is a label-free image analysis, it has potential applications for time-course cell response analysis. Drug profiling based on time dependence will provide detailed information for effectively narrowing down the candidate drugs in vitro.

Based on the morphological analysis of the model neural cells, increased heterogeneity among the cell population can indicate a phenotypic response. Treatment with DHT and PG increased the morphological heterogeneity in the model cells as evidenced by a relative increase in “SD of morphological parameters” in drug-responsive focused pDs. These results suggest the importance of designing cell assays to evaluate multiple phenotypes rather than defining a limited number of phenotypes. This phenomenon exemplifies the challenges associated with evaluating drug responses in neural cells based on a single biomarker or morphological parameter. Increased heterogeneity can also be observed in gene expression in neural stem cells responding to state transition for differentiation. Therefore, in silico FOCUS, which can detect any type of morphological phenotype that appears in response to stimulation, can not only evaluate drug responses but also the quality transitions of other neural cells, which are not readily monitored via biomarker detection.

The flexible applicability of in silico FOCUS motivates us to expand our investigation to other cells. Improved understanding of cellular heterogeneity provides comprehensive insights into cellular responses^32–36^. Single cell analysis has been extensively used to identify unknown cell sub-populations. However, the currently available technologies used to quantitatively profile sub-populations are invasive. Therefore, time-course monitoring of sub-population transition is costly and requires high sample volumes. This technological hurdle inhibits the quality control process in cell therapy products. In particular, this hurdle has served as a bottleneck in understanding the heterogeneity of mesenchymal stem cells with various sub-populations reported to exhibit different therapeutic potencies^32,37,38^. Therefore, condensation and control of such functional sub-populations is a key strategy for controlling cell quality for therapeutic use. However, the identification of specific and robust biomarkers for these objective cell sub-populations is difficult. A non-invasive method to support the in-process monitoring of the cellular population is needed. Sub-population monitoring is also important in other cell types, including induced pluripotent stem cells^9,18^, hematopoietic cells^39^, and programmed cell death protein 1 (PD-1)-expressing T cells^40^. Previously, we had reported the in-process quality monitoring efficacy in mesenchymal stem cells using population profiles^10,16^. However, in silico FOCUS represents another effective method for monitoring the quality of various cell types.

Some “unsuitable cases” apply to in silico FOCUS (flow diagram illustrated in Fig. S7). As in silico FOCUS is developed to effectively analyze “morphologically heterogeneous cells,” it cannot be used to examine “morphologically homogeneous cells or events.” This method was established with the model cells and requires two basic prerequisites. One is the previous knowledge that the target cells exhibit some type of morphological response to increase morphological diversity. The other is the need for a clear positive control, which is the established condition that can provide the biological difference between “target” and “control.” Based on previous studies, our model cells were expected to exhibit morphological responses (extension or elongation). This study also used DHT as a positive control to elicit cellular responses with previously confirmed biological changes. The lack of positive control to validate the in silico FOCUS results makes the interpretation of the outcomes difficult. Moreover, in silico FOCUS is not suitable for use if the target cell type is recalcitrant to morphological changes. Furthermore, effective anomaly iD detection will fail if the drug is cytotoxic and the expected morphological change is simple shrinkage as morphological shrinkage cannot result in the expansion of the Mahalanobis distance. Therefore, in silico FOCUS is effective at detecting “morphology expanding effects” but not at analyzing “morphology shrinking” cytotoxic effects.

The balancing of sensitivity and reliability is challenging when focusing on minor sub-populations using image analysis. The quality of data extracted from the image must be controlled as the number of measurable cells decreases in the image, which increases the risk of capturing non-reliable events. Compared with the fluorescently labeled images, label-free images are low-contrast with detailed focus accuracy. Thus, cells in the image can be readily lost due to image processing issues (such as inaccurate focus triggered by meniscus, bubble on the liquid surface, distortion of well-plate, and scratches on the bottom). The field-of-view bias is an unwanted bias that can significantly skew the results of label-free morphological analysis. However, solutions to such influences have rarely been discussed in the previous label-free image analysis studies. Data processing after cytometric measurements, such as data pooling, pD creation with bootstrap, and in silico FOCUS analysis for enriching anomaly iDs effectively stabilized the data extracted from images. Thus, in silico FOCUS analysis can be postulated to enable robust analysis of other label-free images.

Although machine learning is a powerful tool to accelerate image-based cell evaluation, several issues must be considered. The constructed model should be explainable for ensuring the associated prediction accuracy, which remains a major point of contention in artificial intelligence^41^. As data volume cannot be readily increased in cell-based drug screening assays, high-performance machine learning models are associated with the risk of overfitting non-robust parameters. Meanwhile, the iDs and pDs obtained from in silico FOCUS describe morphological information that can be confirmed using the raw image. Therefore, the reliability of the classification model with the employed parameters can be readily confirmed. PCA and clustering analysis (without using machine learning) demonstrated that focused pD obtained from in silico FOCUS are stable descriptors for characterizing drug-responsive cells. In contrast to examining only the classification performance of machine learning models, these analyses support the reliability of the model. The use of a balanced portion of training data represents an additional critical point^10,42,43^. Equivalent quantities and qualities of both types of training data (“control” and “target”) must be prepared to obtain a robust classification model. Extreme imbalances in the training data adversely affect the model robustness. However, all types of morphologies for all drug candidates cannot be characterized during drug screening. The introduction of the MT method enabled us to overcome these limitations. The MT method, an anomaly discrimination method, has been used for manufacturing quality controls in product manufacturing^30,31^. This simple algorithm requires only the “normal status” data for model training as a unit space and supports the detection of anomalies with minimal calculation costs. Therefore, both model training and anomaly prediction require only a few seconds, which makes it suitable for real-time image-based phenotypic screening. Thus, this anomaly discrimination concept reduces the data preparation cost and effort for identifying “target” cells. Consequently, in silico FOCUS does not require prior experiments to examine and define the target sub-population of drug-responding cells to create a high-performance image-based classification model. Moreover, as the concept of anomaly iD selection is defined as “anomaly ranking of the top 50% of the population,” it also minimizes the need for defining a threshold between the unit space and the anomalies required in the MT method. Therefore, no special parameter tuning is required when extrapolating the in silico FOCUS method to other cell types.

Thus, the in silico FOCUS workflow developed in this study is a feasible and robust method for sensitive sub-population evaluation based on label-free image analysis even for morphologically heterogeneous cells. This simple method overcomes limitations associated with conventional machine learning models for analyzing cell images and has various applications.

## Methods

### Cell culture

Described in the supplementary material.

### Mitochondrial activity assay and metabolism measurement

Described in the supplementary material.

### Image acquisition

Described in the supplementary material.

### Morphological analysis

Morphological analysis is a combination of image processing, data processing, and analysis (Fig. S3). Image processing begins with iD measurement. All images were processed using CL-Quant (Nikon Corporation), following a previously reported procedure^16^. Briefly, five original image processing methods were designed to recognize cells as objects in the image using the following steps: (1) background adjustment, (2) texture enhancement, (3) cell recognition, (4) noise removal, and (5) filling in recognized regions (Fig. S8a). Next, the recognized objects (mostly cells) were measured using 14 morphological parameters (Table S2). The data comprised object labels tagged with 14 morphological parameter sets defined as “iD.” A noise reduction algorithm (Japan patent no. 5745290) was used to clean the incorrectly recognized objects in the background from among the iD (Fig. S8b). The clean iD were then pooled in the data pool to minimize experimental bias from certain fields-of-view or wells. iD from replicate wells (omitting wells for which appropriate focus was not achieved) were accumulated for a single experimental condition category. For data processing, pD were created by summarizing iD to the described measured cell populations. To summarize the iD, the mean and SD were calculated (Table S2). Thus, “pD” indicate the data with sample labels tagged with the mean and SD of the morphological parameters. Among the 28 parameters (14 morphological parameters × [mean and SD]), only “Length_width_ratio_SD” was eliminated as it was unstable with a high CV (more than twice as high as other parameters). To minimize the experimental bias, bootstrapping was introduced to produce multiple pD from the iD pool. Random sampling of 100 iD was repeated 10 times to permit overlap and produce 10 pD per condition. To compare the effect of data pooling and bootstrapping, the data were characterized into the following three types of pD: (1) raw pD, calculated from iD without data pooling and bootstrapping, which included the experimental bias; (2) pD with bootstrapping (b-pD), calculated from iD repeatedly collected by bootstrap sampling from the data pool; (3) focused pD, calculated from iD repeatedly collected by bootstrap sampling from the control iD pool or iD pool enriched with anomaly iD using in silico FOCUS (focused iD).

### In silico FOCUS data processing

In this method, “control” and “target” were defined as the states without and with the drug, respectively. This morphology-based cytometry process includes the following four steps (Fig. 1b): step 1, collection of iD from the “control” and “target (with drug)” groups; step 2, all control iD were used for training the “unit space” based on the discrimination concept from the MT method; step 3, anomaly discrimination of target iD using the MT method. The Mahalanobis distance from the centroid of the unit space (obtained in step 2) to every individual target iD was calculated using 14 morphological parameters. Target iD were ranked based on their distance from the centroid of the unit space; step 4, pD were calculated. From the distance ranking of the target iD, the top 50% of the total iD population were collected as anomaly iD. The percentage of these data in pD reflected the morphological changes in the drug-responding cells. This percentage iD collection is one of the important hyperparameters in this method and can be optimized to tune the in silico FOCUS algorithm to other cell types or responses. Bootstrap sampling (100 iD repeatedly resampled 10 times, permitting overlaps) was employed for the anomaly iD to produce 10 focused pD, representing the target. For focused pD representing the control, the same bootstrap sampling was employed for the pool of all control iD. Data processing was performed using R (version 3.4.1) (R Development Core Team, https://www.r-project.org/). For analysis, pD were used for PCA, hierarchical clustering, and machine learning to construct the phenotype classification model.

### Unsupervised analysis of the morphological profile data

Described in the supplementary material.

### Evaluation of in silico FOCUS effect with statistical tests

To quantify the effect of in silico FOCUS, the following two criteria were evaluated between b-pD and focused pD: criteria 1, variance of morphological parameters among pD; criteria 2, significance of morphological differences for each morphological parameter. Criteria 1 (Fig. S9a) indicates the variance in the distribution of 27 CVs from 27 morphological parameters. Each CV indicates the diversity of single morphological parameters among 10 pD. If 10 pD are stably created, each morphological parameter among the 10 pD should indicate low CV, resulting in the distribution of CVs with low mean and small SD. Welch's *t*-test adjusted by Bonferroni correction was applied to test the differences in CVs between the following conditions: “raw pD vs. focused pD” and “b-pD vs. focused pD” (Fig. 2k), “b-pD vs. focused pD” (Figs. S4b and S5c). The significant decrease in these comparisons indicated that pD were stable. Criteria 2 (Fig. S9b) is the number of morphological parameters exhibiting significant differences individually in control vs. target comparison. If there is a stable contribution of morphological parameters in the classification of phenotypes, these descriptors are expected to classify phenotypes even individually. For comparing control and target differences, the distribution of 10 morphological parameters from 10 pD were tested in each morphological parameter. Dunnett’s test was used for analysis. The significant morphological parameter (*p* < 0.05) was marked in the matrix (Figs. 2l, S4c, and S5d). Compared pD pairs are illustrated in Fig. S9b.

### Sample size effect evaluation

Described in the supplementary material.

### Construction of the classification model

Described in the supplementary material.

## Supplementary Information

Supplementary Information.

## Data Availability

The datasets generated during and/or analyzed during the study are available from the corresponding author on reasonable request.
