# Supplementary material for: Label-free morphological sub-population cytometry for sensitive phenotypic screening of heterogenous neural disease model cells
Source: Sci Rep. 2022 Jun 16;12:9296. doi: 10.1038/s41598-022-12250-0 (PMC9203459; doi:10.1038/s41598-022-12250-0)
Supplement: Supplementary file 1 — Supplementary Information. [file 41598_2022_12250_MOESM1_ESM.pdf]

Label-free morphological sub-population cytometry for sensitive phenotypic screening of heterogenous neural disease model cells

Yuta Imai[1], Madoka Iida[2], Kei Kanie[1], Masahisa Katsuno[2,3,4,5], Ryuji Kato[1,3,5,*]

[1]Department of Basic Medicinal Sciences, Graduate School of Pharmaceutical Sciences, Nagoya University, Tokai National Higher Education and Research System, Furocho, Chikusa-ku, Nagoya, Aichi 464-8601, Japan

[2]Department of Neurology, Nagoya University Graduate School of Medicine, Tokai National Higher Education and Research System, 65 Tsurumai-cho, Showa-ku, Nagoya, Aichi 466-8550, Japan

[3]Institute of Nano-Life-Systems, Institutes of Innovation for Future Society, Nagoya University, Tokai National Higher Education and Research System, Furocho, Chikusa-ku, Nagoya, Aichi 464-8601, Japan

[4]Department of Clinical Research Education, Nagoya University Graduate School of Medicine, Tokai National Higher Education and Research System, 65 Tsurumai-cho, Showa-ku, Nagoya, Aichi 466-8550, Japan

[5]Institute of Glyco-core Research (IGCORE), Nagoya University, Tokai National Higher Education and Research System, Furocho, Chikusa-ku, Nagoya, Aichi 464-8601, Japan

Methods

Cell culture

The SBMA model cell lines AR-24Q (healthy model) and AR-97Q (disease model) were established by fusing the motor neuron-enriched embryonic mouse spinal cord cells with the mouse neuroblastoma NSC34 cells (kindly provided by N. R. Cashman, University of British Columbia, Vancouver, Canada), as reported in our previous work[22]. Both cell lines were genetically modified to stably express human full-length ARs with different poly-glutamine repeats (regular repeats (24Q) or aberrant repeats (97Q)). Dihydrotestosterone (DHT; Tokyo Chemical Industry Co., Ltd., Tokyo, Japan), a natural ligand of AR that stimulates downstream signaling, was used to generate disease/healthy phenotypes in the model cells. Iida *et al*. demonstrated that the PPARγ ligand pioglitazone (PG) effectively rescued the SBMA phenotype under DHT stimulation[22]. Therefore, PG (Cayman Chemical Co, Ann Arbor, MI, USA) was used as the model drug to rescue the disease phenotype in DHT-stimulated AR-97Q cells. DHT was first dissolved in ethanol. Four concentrations (1, 5, 10, and 20 nM) of DHT were prepared in phosphate-buffered saline (PBS). PG was first dissolved in dimethyl sulfoxide (DMSO). Two concentrations (0.1 and 1 μM) of PG were prepared in PBS. The cells were incubated with PG along with 10 nM DHT. Both model cell types (Passage 7) were maintained in Dulbecco's Modified Eagle's Medium (DMEM) (Nacalai Tesque, Kyoto, Japan) supplemented with 10% fetal bovine serum (FBS) (Nichirei Biosciences Inc., Tokyo, Japan) and 1% penicillin-streptomycin (PS) (Nacalai Tesque) at 37 °C and 5% CO_2. Neural differentiation was induced by decreasing the FBS concentration to 1% after two days of growth. Drug administration accompanied by morphological evaluation was initiated on the first day of differentiation.

Mitochondrial activity assay

Mitochondrial activity was assayed by measuring the mitochondrial membrane potential using MitoTracker™ Orange CMTMRos (Invitrogen, Carlsbad, CA, USA). The cells were incubated with 100 nM of dye for 30 min at 37 °C in serum-free DMEM and washed with pre-warmed serum-free DMEM. The fluorescence intensity of the samples was measured using Fluoroskan Ascent FL (Thermo LabSystems Inc, PA, USA) at excitation and emission wavelengths of 544 and 590 nm, respectively. In the same well, viable cells were stained with Calcein-AM (Dojindo Laboratories Co., Ltd., Kumamoto, Japan) for 30 min and analyzed with Fluoroskan Ascent FL (Thermo LabSystems Inc) at excitation and emission wavelengths of 384 and 544 nm, respectively. Mitochondrial activity values were normalized to the Calcein-AM values.

Metabolism measurement

Metabolic measurements (glucose, lactate, glutamic acid (Glu), and glutamine (Gln)) were performed using BioProfile FLEX2 (Nova Biomedical K.K., Tokyo, Japan). Under each culture condition, 400 μL of the culture supernatant was analyzed. Supernatants were collected at days 2 and 3 post-seeding. The consumption or production rate of each component was determined.

Image acquisition

Phase-contrast microscopy images were acquired for cells grown in 24-well plates using an automatic cell image acquisition system (BioStation CT, Nikon Corporation, Tokyo, Japan). Cells were seeded at a density of 2000 cells/cm^2 in triplicate. The cells were cultured for 2 days and allowed to undergo neural differentiation for 2 days. The images were captured at 4× magnification (single point per well, covering 4 mm^2; 1000 pixels^2/image). For each condition, 3–6 replicate images were collected. Each image was set in the center of the well to have minimum disturbance of meniscus and included more than 200 cells.

Unsupervised analysis of the morphological profile data

Morphological similarities among individual data (iDs) and population data (pDs) were visualized using principal coordinate analysis (PCA). For data-segmented visualization, all data were plotted once in the same PCA. The weights of all parameters were saved and used for plotting individual categories of data. Hierarchical clustering (using correlation coefficient with average linkage) was used for categorizing objective experimental conditions, followed by morphological category implementation. To reduce the bias of highly correlated parameters in the clustering, the mean and standard deviation (SD) of the texture parameters were eliminated. All analyses and visualizations were performed using R (version 3.4.1) (R Development Core Team, https://www.r-project.org/).

Sample size effect evaluation

From the control and DHT (20 nM) response condition data, random sampling was repeated to generate 50 datasets with 10, 50, 100, 150, 200, and 250 iDs. *In silico* FOCUS was applied to the DHT response condition data using the unit space trained with the control data. After the calculation of Mahalanobis distances for every iD in target cells, the Mahalanobis distance distribution data between the control and the target were

compared using the Welch's *t*-test. Among the 50 trials of datasets, the numbers of significances to discriminate target iDs were counted and indicated as the percentage.

Construction of the classification model

To construct the phenotype classification model, the following three types of phenotype data were used: disease data, 20 pDs from disease phenotype of AR-97Q cells responding to DHT; rescued data, 40 pDs from rescued phenotypes of AR-97Q cells responding to PG (1 μM) with DHT; healthy data, 20 pDs from healthy phenotypes of AR-24Q cells responding to DHT. Model A was trained to classify disease and rescued data, while Model B was trained to classify disease and healthy data. The performances of the two models were validated by leave-(all samples from the same well)-out cross-validation (modification of leave-one-out, leaving "pDs from the same well" out). For blind test data for model A, healthy data were used. Meanwhile, rescued data were used for blind test data for model B. LASSO was used for the discrimination model, which was coded using R (version 3.4.1).

Figures

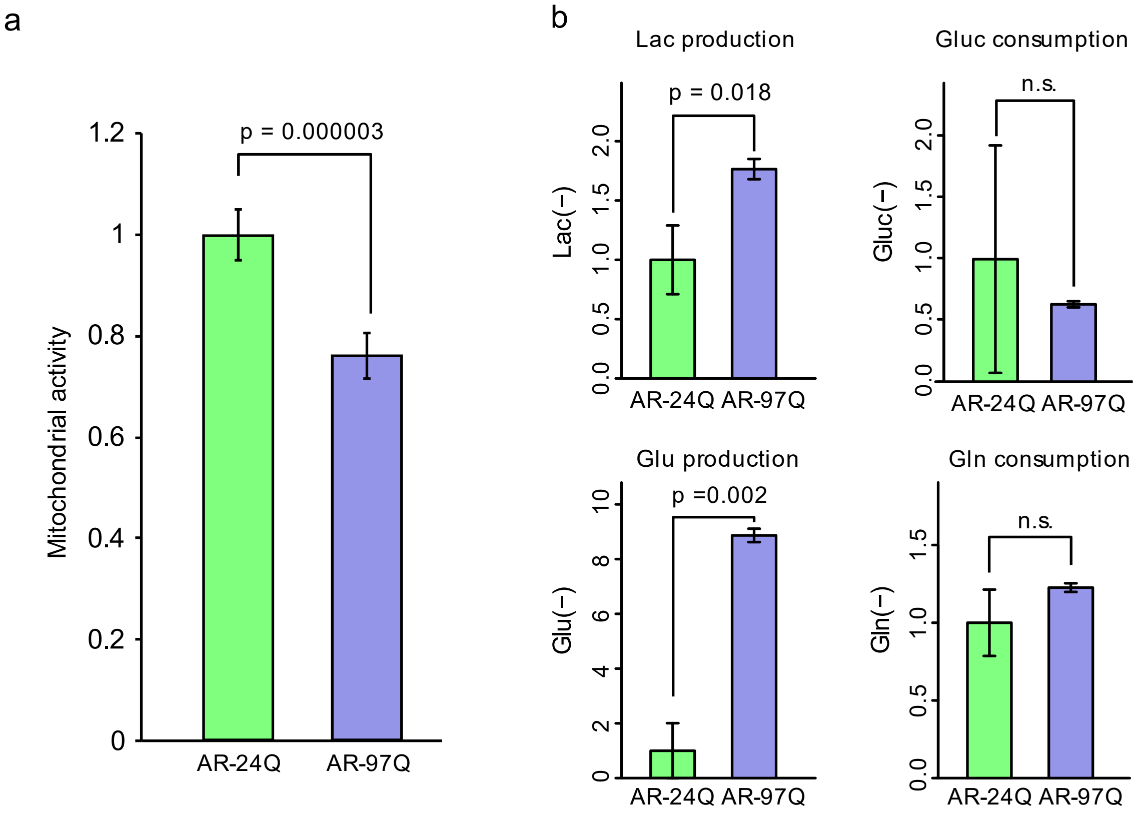

Fig. S1 Discrimination performance of the bulk assay on 20,000–30,000 model cells. (**a**) Comparison of mitochondrial activity. (N = 6) (**b**) Comparison of measured metabolites; Lactate (Lac) and glucose (Glu) production (left), Gluc and glutamine (Gln) consumption (right). (N = 3) Bar plots indicate mean, while error bars indicate standard deviation. n.s. indicates non-significance. All illustrations created by Adobe Illustrator 24.1.1 (https://www.adobe.com/jp/products/illustrator.html).

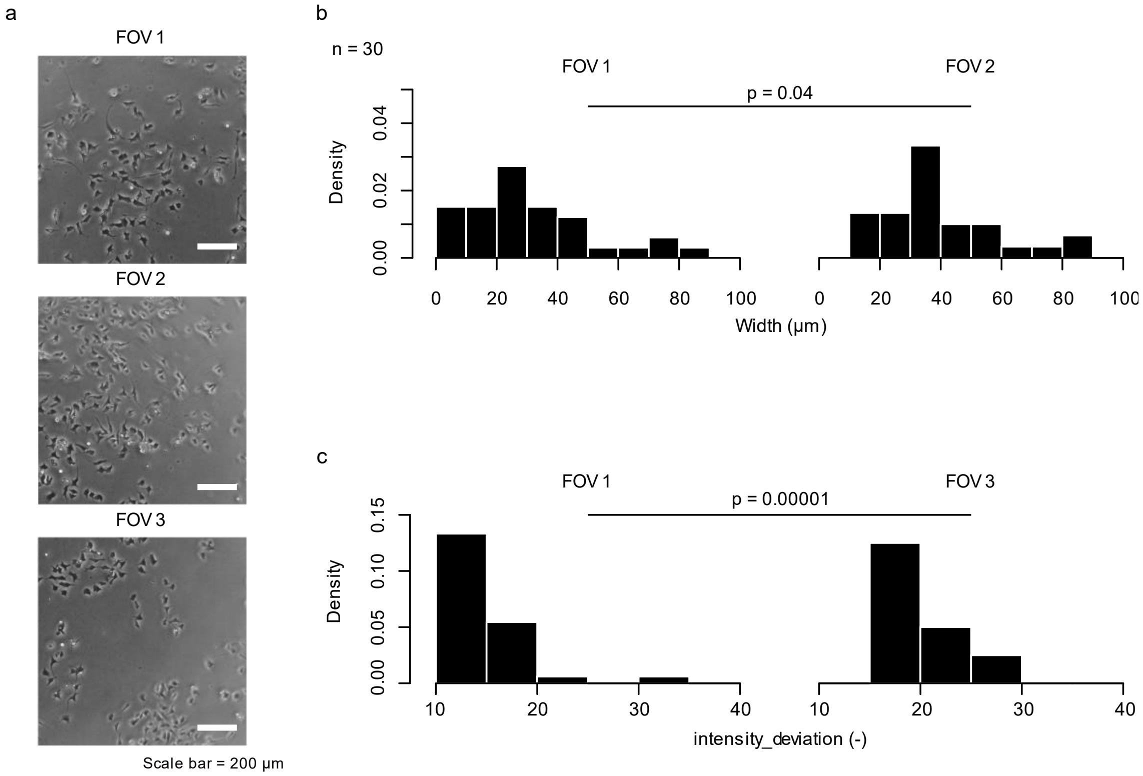

Fig. S2 Representative data for showing the influence of experimental bias on morphological data.

(**a**) Three fields of view (FOVs) indicating the different distribution of cells after seeding the same density of cells in the well. (**b**–**c**) Morphological distributions from each FOV (b, width; c, intensity deviation). The Kolmogorov-Smirnov test revealed that two FOVs are significantly different although they represented the same experimental condition (N = 30). All illustrations created by Adobe Illustrator 24.1.1 (https://www.adobe.com/jp/products/illustrator.html).

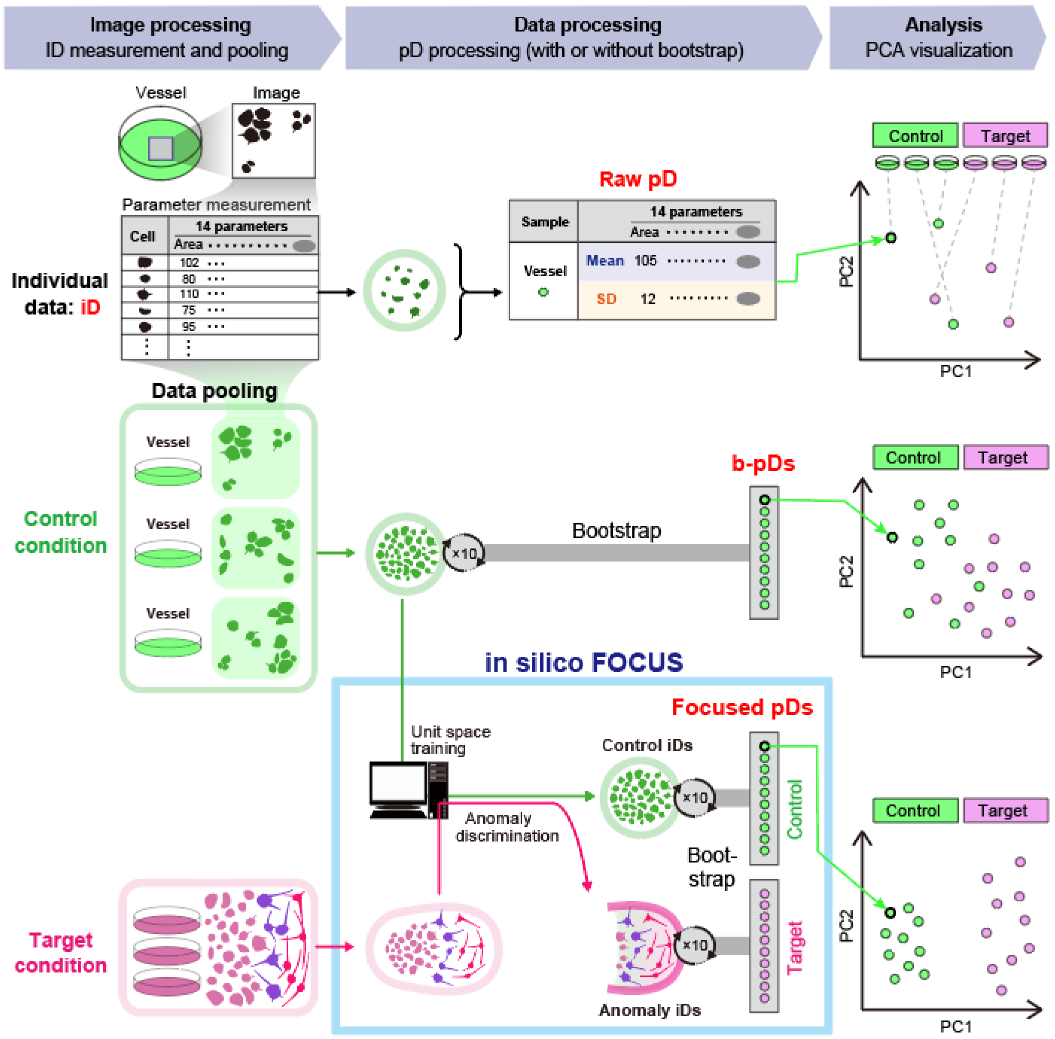

Fig. S3 Schematic illustration of workflow for morphological analysis.

Morphological analysis comprised image processing, data processing, and analysis. (Image processing) From the obtained images, single cells are identified and their morphological parameters are measured to generate individual data (iDs). iDs are pooled *in silico* to reduce the experimental bias. (Data processing) From the pool, iDs are collected to generate pD and evaluate the mean and standard deviation (SD) of iDs. The following three types of pDs were compared in this study: raw pDs, calculated from iDs collected from iD pool of each vessel; pDs with bootstrapping (b-pDs), calculated from iDs collected from pooled iDs using bootstrap; focused pDs, calculated from iDs collected from control pooled iDs or target iDs enriched by anomaly iDs using in silico featured-objects concentrated by anomaly discrimination from unit space (*in silico* FOCUS) analysis with bootstrap. (Analysis) The pDs were used for unsupervised and supervised machine learning. In this illustration, principal coordinate analysis (PCA) is

shown as an example. In raw pDs, one sample indicates one vessel, whereas in b-pDs and focused pDs, 10 dots indicate the same condition. All illustrations created by Adobe Illustrator 24.1.1 (https://www.adobe.com/jp/products/illustrator.html).

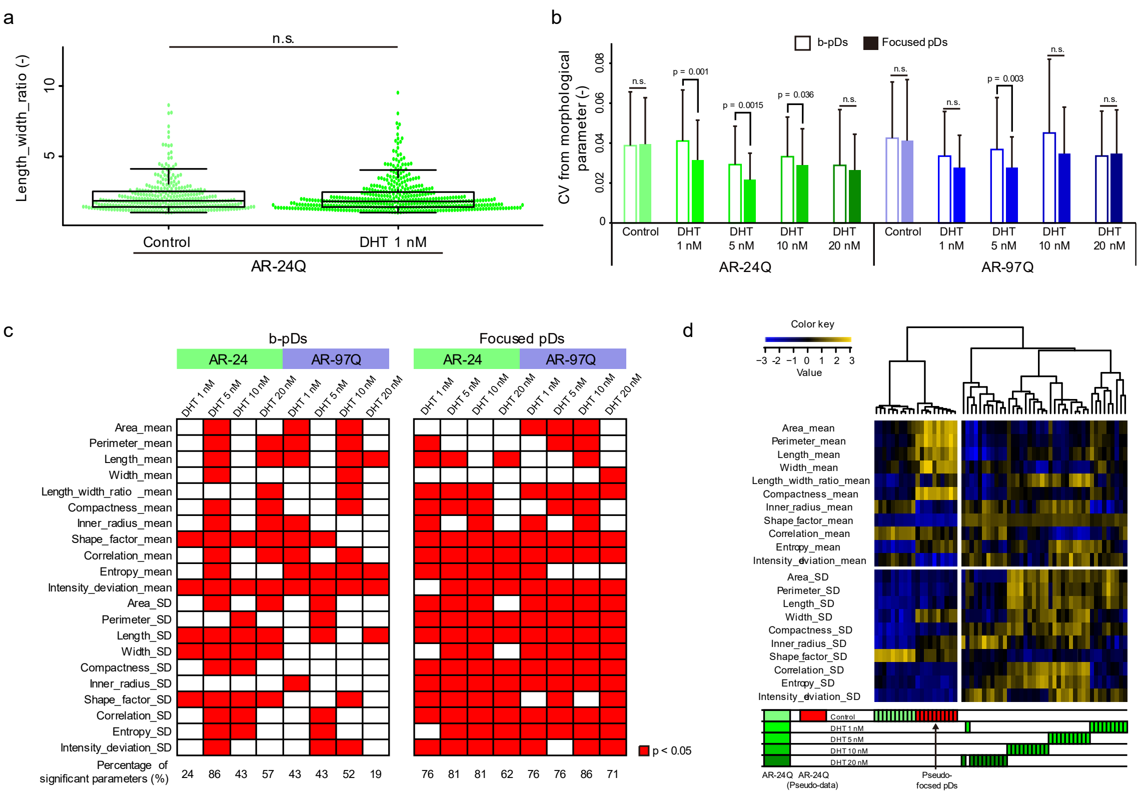

Fig. S4 Evaluation of the phenotypic responses of model cells to dihydrotestosterone (DHT).

(**a**) Morphological distribution of control and DHT (1 nM)-stimulated AR-24Q cells described by length_width_ratio. n.s. indicates non-significance (N = 300). (**b**) Evaluation the effect of *in silico* featured-objects concentrated by anomaly discrimination from unit space (*in silico* FOCUS) using the variance of morphological parameters among population data (pDs). In each condition, the stability of 10 pDs with bootstrapping (b-pDs) and 10 focused pDs were compared with the 27 coefficient of variations (CVs), each representing the variance of single morphological parameters. $p < 0.05$ is indicated in the plot (N = 27). The bar plots indicate the mean of 27 CVs, while error bars indicate the standard deviation (SD). If the distribution of CVs shows a low mean with a small SD, it indicates that 10 pDs are stably created. (**c**) Evaluation of *in silico* FOCUS effect using the significant morphological parameters ($p < 0.05$) between control and DHT-stimulated cells (N = 10). (d) Clustering of pDs from *in silico* FOCUS (focused pDs) with pseudo-focused pDs obtained from control data used as target data (red label). All illustrations created by Adobe Illustrator 24.1.1 (https://www.adobe.com/jp/products/illustrator.html).

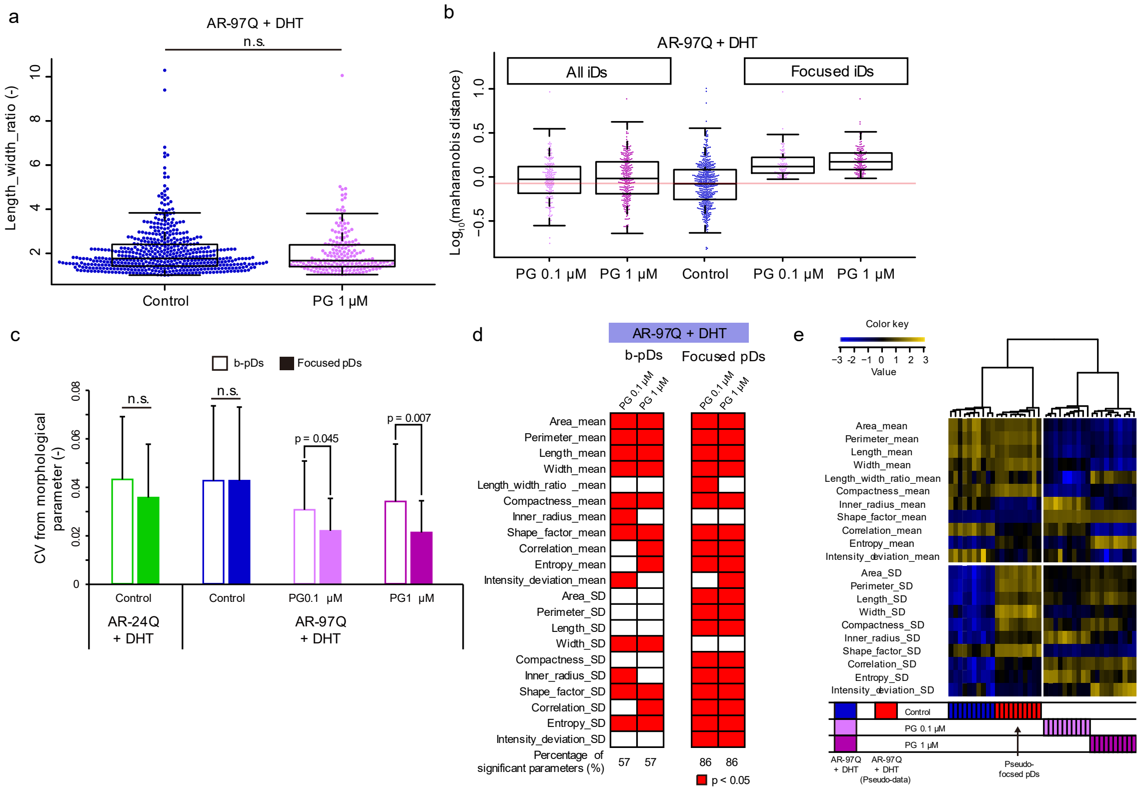

Fig. S5 Evaluation of the phenotypic responses of model cells to pioglitazone (PG).

(**a**) Morphological distribution of control and pioglitazone (PG; 1 μM) described by length_width_ratio. n.s. non-significance (N = 300). (**b**) Evaluation of the effect of *in silico* featured-objects concentrated by anomaly discrimination from unit space (*in silico* FOCUS) using the variance of morphological parameters among population data (pDs). Distribution of Mahalanobis distances of individual data (iDs) (all iDs, control, and anomaly iDs from *in silico* FOCUS) from the centroid of the unit space defined by control iDs. Box plot (median at the center, and the 25 and 75 percentiles at the end) with whiskers (standard deviation (SD)) overlaid on the beeswarm plot. The red line indicates the mean of control iDs. (**c**) Evaluation of *in silico* FOCUS effect using the variance of morphological parameters among pDs. In each condition, the stability of 10 pDs with bootstrap (b-pDs) and 10 focused pDs was compared with the 27 coefficient of variants (CVs), each representing the variance of single morphological parameter. $p < 0.05$ is indicated in the plot (N = 27). The bar plots indicate the mean of 27 CVs, while error bars indicate SD. If the distribution of CVs shows a low mean with a small SD, it indicates that 10 pDs are stably created. (**d**) Evaluation of *in silico* FOCUS effect using the significant morphological parameters ($p < 0.05$) between control and dihydrotestosterone (DHT)-stimulated cells (N = 10). (**e**) Clustering of pDs from *in silico* FOCUS (focused pDs) with pseudo-focused pDs obtained from control data used as target data (red label).

All illustrations created by Adobe Illustrator 24.1.1 (https://www.adobe.com/jp/products/illustrator.html).

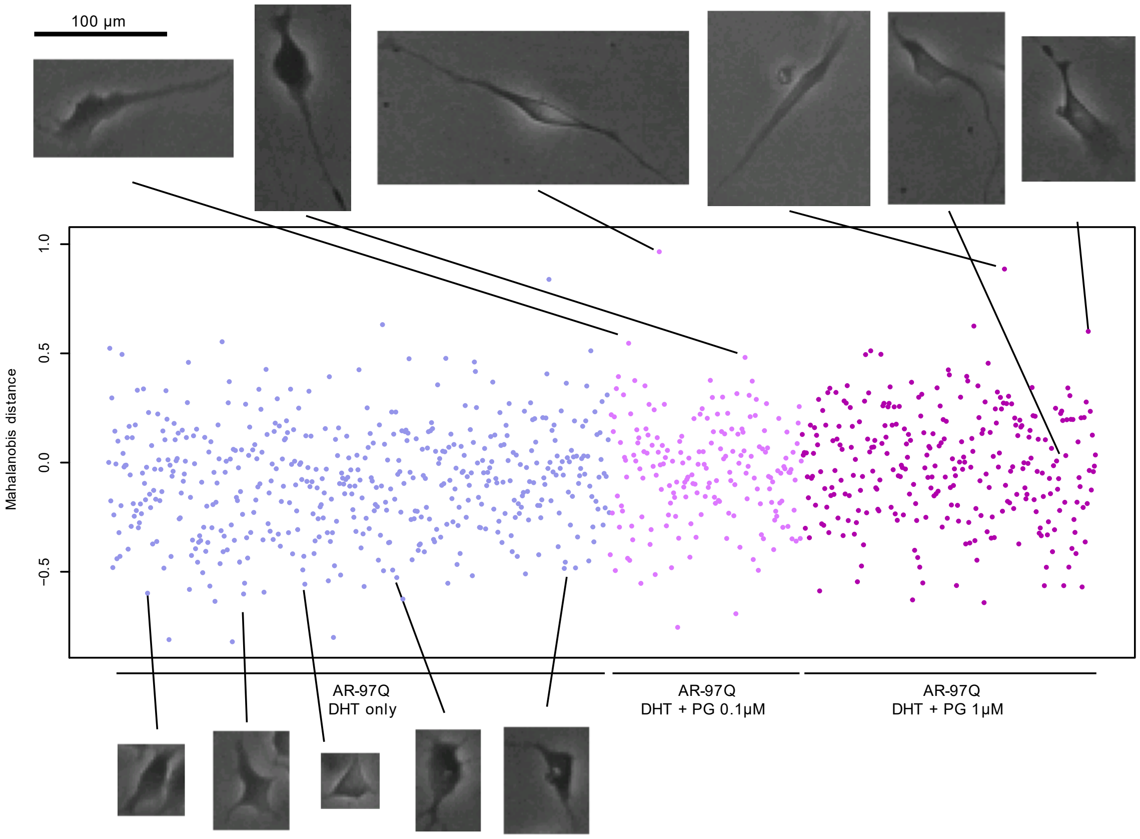

Fig. S6 Raw data distribution of individual data (iDs) in control (dihydrotestosterone (DHT)-treated) in unit space and 0.1 μM pioglitazone (PG)-treated (with DHT), and 1 μM PG-treated (with DHT) AR-97Q cells and representative cell morphology images. The images at the top of the graph show cells with a large Mahalanobis distance from the centroid of the unit space. The image at the bottom of the graph shows cells with the most typical morphologies found in the control cell population. All illustrations created by Adobe Illustrator 24.1.1 (https://www.adobe.com/jp/products/illustrator.html).

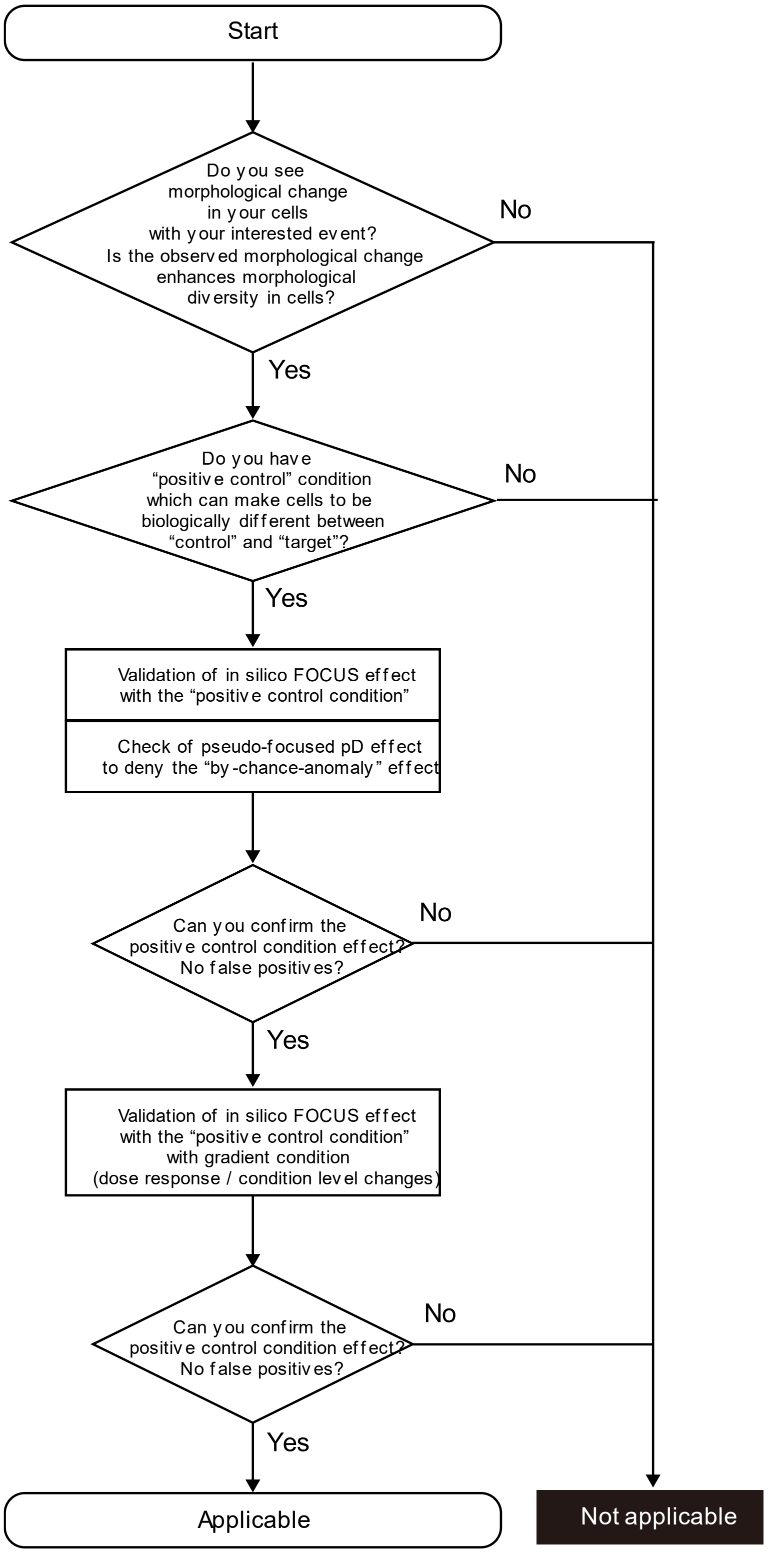

Fig. S7 Flow-diagram to check the applicability of *in silico* featured-objects concentrated by anomaly discrimination from unit space (*in silico* FOCUS) analysis. All illustrations created by Adobe Illustrator 24.1.1 (https://www.adobe.com/jp/products/illustrator.html).

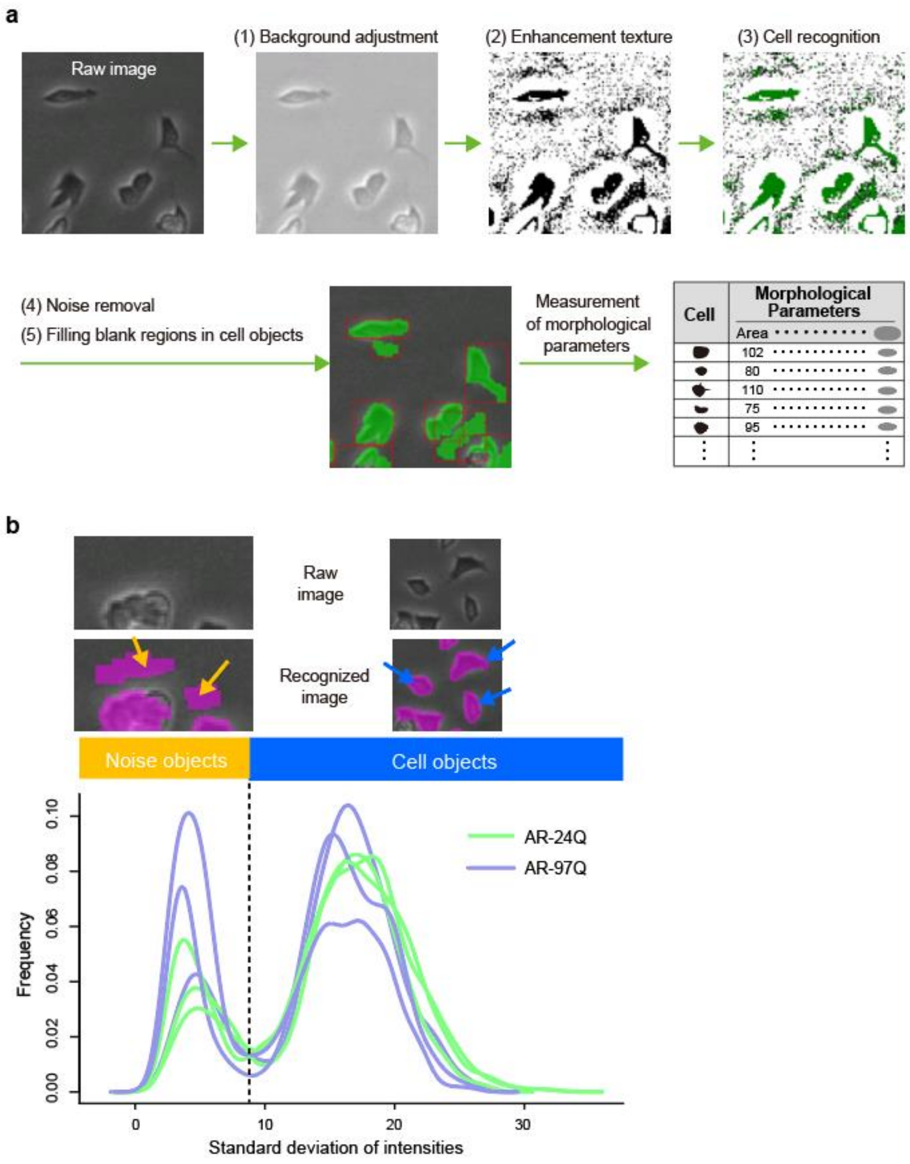

Fig. S8 Schematic illustration of image processing.

(**a**) Pipeline of image processing. (**b**) Concept of the noise reduction algorithm. Briefly, the algorithm performs *in silico* morphological cytometric measurements and effectively collects "cell objects" from all the recognized objects in the image by thresholding them with standard deviation of intensities in each object. The figure illustrates the distribution of raw data of recognized objects from three wells containing AR-24Q and AR-97Q cells and the effect of discriminating noise objects and cell objects with threshold (dotted vertical line). All illustrations created by Adobe Illustrator 24.1.1 (https://www.adobe.com/jp/products/illustrator.html).

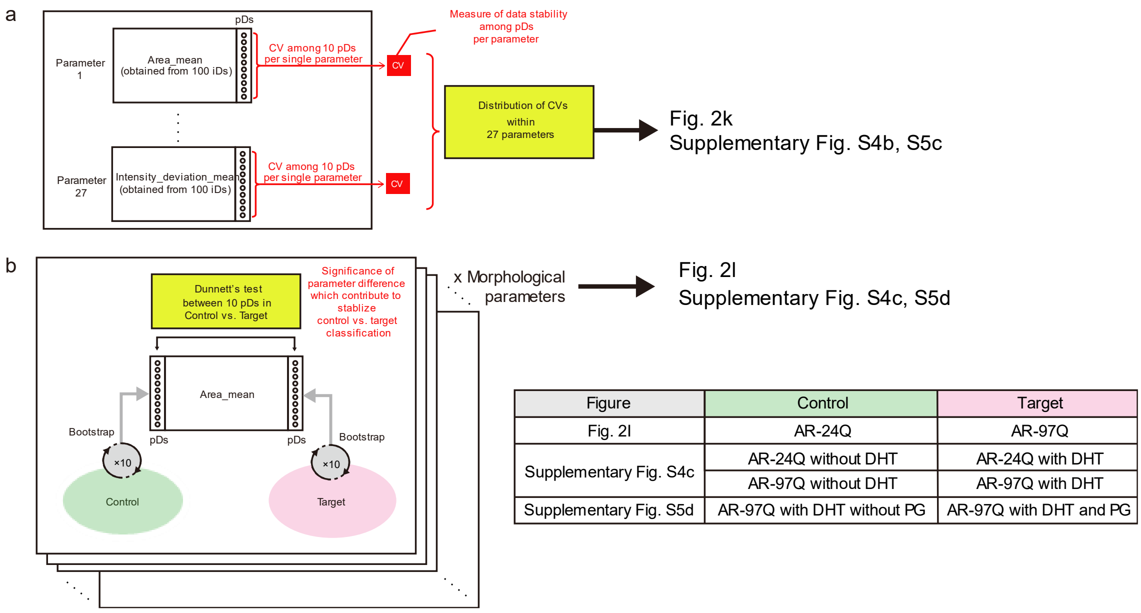

Figure	Control	Target
Fig. 2l	AR-24Q	AR-97Q
Supplementary Fig. S4c	AR-24Q without DHT	AR-24Q with DHT
	AR-97Q without DHT	AR-97Q with DHT
Supplementary Fig. S5d	AR-97Q with DHT without PG	AR-97Q with DHT and PG

Fig. S9 Schematic illustration describing the calculation concept for the criteria for evaluating the effect of *in silico* featured-objects concentrated by anomaly discrimination from unit space (*in silico* FOCUS).

(**a**) Calculation concept of criteria 1 (variance of morphological parameters among population data (pDs)). Each coefficient of variation (CV) indicates the diversity of a single morphological parameter among 10 pDs. All 27 CVs are used to show the distribution of CVs in Figs. 2k, S4b, and S6c. (**b**) Calculation concept of criteria 2 (significance of morphological differences in each morphological parameter). For comparing control and target differences in each morphological parameter, the distribution of ten morphological parameters from ten pDs were tested and repeated with all parameters. The significant ($p < 0.05$) morphological parameter was colored in the matrix (Figs. 2l, S4c, and S6d). List of compared pD pairs (control vs. target) is also indicated. All illustrations created by Adobe Illustrator 24.1.1 (https://www.adobe.com/jp/products/illustrator.html).

Table S1. *In silico* FOCUS analysis of the effect of sample size in collected iDs indicated by the significant difference in the percentage of discriminating target iD

			AR-24Q DHT 20 nM Target Sample size (cells)					
			10	50	100	150	200	250
AR-24Q Control (Unit space)	Sample size (cells)	10	6f6%	98%	**100%**	**100%**	**100%**	**100%**
		50	24%	86%	**100%**	**100%**	**100%**	**100%**
		100	26%	80%	**100%**	**100%**	**100%**	**100%**
		150	16%	68%	**100%**	**100%**	**100%**	**100%**
		200	20%	88%	**100%**	**100%**	**100%**	**100%**
		250	20%	74%	**98%**	**100%**	**100%**	**100%**
		300	14%	80%	**98%**	**100%**	**100%**	**100%**

All the percentages indicate the significant discrimination of the target from control evaluated using Welch's *t*-test in 50 trials. iD, individual data; *in silico* FOCUS, *in silico* analysis of featured-objects concentrated by anomaly discrimination from unit space; DHT, dihydrotestosterone.

Table S2. Morphological parameters to describe iD and pD in this work

Parameter number	Indicated name	Individual name	Statistics	Definition	Note
1	Area_mean	Area	Mean (mean of collected iDs)	Total pixels in the recognized cell region.	
2	Perimeter_mean	Perimeter		The arc length of recognized cell region.	
3	Length_mean	Length		Long axis of bounding rectangle covering cell.	
4	Width_mean	Width		Short axis of bounding rectangle covering cell.	
5	Length_width_ratio_mean	Length_width_ratio		Length/Width	
6	Compactness_mean	Compactness		(Perimeter)^2/Area	
7	Inner_radius_mean	Inner_radius		Radius of inscribed circle from the centroid of cell area.	
8	Shape_factor_mean	Shape_factor		4π(Area)/(Perimeter)^2	
9	Correlation_mean	Correlation		Gray-Level Co-occurrence Matrix (GLCM) of cell region. GLCM = M Correlation $= \sum_{i,j} \frac{(i-\mu_x)(j-\mu_y) M_{i,j}}{\sigma_x \sigma_y}$	

			$\mu_x = \sum_{i,j} jM_{i,j}$<br>$\mu_y = \sum_{i,j} iM_{i,j}$	
10	Entropy_mean	Entropy	Gray-Level Co-occurrence Matrix (GLCM) of cell region.<br>$GLCM = M$<br>$Entropy = -\sum_{i,j} M_{ij} \log M_{i,j}$	
11	Energy_mean	Energy	Gray-Level Co-occurrence Matrix (GLCM) of cell region.<br>$GLCM = M$<br>$Energy = \sum_{i,j} M_{i,j}^2$	
12	Homogony_mean	Homogony	Gray-Level Co-occurrence Matrix (GLCM) of cell region.<br>$GLCM = M$<br>$Homogony = \sum_{i,j} \frac{M_{i,j}}{1 + \lvert i-j \rvert}$	This parameter was omitted in the clustering visualization
13	Intertia_mean	Inertia	Gray-Level Co-occurrence Matrix (GLCM) of cell region.<br>$GLCM = M$<br>$Inertia = \sum_{i,j} (i-j)^2 M_{ij}$	This parameter was omitted in the clustering visualization
14	Intensity_deviation_mean	Intensity_deviation	Standard deviation of intensities of pixels in cell region.	

15	Area_SD	Area	SD (SD of collected iDs)	Total pixels in the recognized cell region.	
16	Perimeter_SD	Perimeter		The arc length of recognizaed cell region.	
17	Length_SD	Length		Long axis of bounding rectangle covering cell.	
18	Width_SD	Width		Short axis of bounding rectangle covering cell.	
19	Length_width_ratio_SD	Lendth_width_ratio		Length/Width	This parameter was finally deleted in the pD, because of its high noise.
20	Compactness_SD	Compactness		(Perimeter)^2/Area	
21	Inner_radius_SD	Inner_radius		Radius of inscribed circle from the centroid of cell area.	
22	Shape_factor_SD	Shape_factor		4π(Area)/(Perimeter)^2	
23	Correlation_SD	Correlation		Gray-Level Co-occurrence Matrix (GLCM) of cell region. $\mathrm{GLCM} = \mathrm{M}$ $\mathrm{Correlation} = \sum_{i,j} \frac{(i-\mu_x)(j-\mu_y)M_{i,j}}{\sigma_x \sigma_y}$ $\mu_x = \sum_{i,j} jM_{i,j}$	

			$\mu_y = \sum_{i,j} iM_{i,j}$	
24	Entropy_SD	Entropy	Gray-Level Co-occurrence Matrix (GLCM) of cell region.<br>$GLCM = M$<br>$Entropy = -\sum_{i,j} M_{ij} \log M_{i,j}$	
25	Energy_SD	Energy	Gray-Level Co-occurrence Matrix (GLCM) of cell region.<br>$GLCM = M$<br>$Energy = \sum_{i,j} M_{i,j}^2$	
26	Homogony_SD	Homogony	Gray-Level Co-occurrence Matrix (GLCM) of cell region.<br>$GLCM = M$<br>$Homogony = \sum_{i,j} \frac{M_{i,j}}{1 + \|i-j\|}$	This parameter was omitted in the clustering visualization
27	Inertia_SD	Inertia	Gray-Level Co-occurrence Matrix (GLCM) of cell region.<br>$GLCM = M$<br>$Inertia = \sum_{i,j} (i-j)^2 M_{ij}$	This parameter was omitted in the clustering visualization
28	Intensity_deviation_SD	Intensity_deviation	Standard deviation of intensities of pixels in cell region.	

iD, individual data; pD, population data.
